# High Coke-Resistance Pt/Mg_1-x_Ni_x_O Catalyst for Dry Reforming of Methane

**DOI:** 10.1371/journal.pone.0145862

**Published:** 2016-01-08

**Authors:** Faris A. J. Al-Doghachi, Aminul Islam, Zulkarnain Zainal, Mohd Izham Saiman, Zaidi Embong, Yun Hin Taufiq-Yap

**Affiliations:** 1 Catalysis Science and Technology Research Centre, Faculty of Science, University Putra Malaysia, 43400, UPM, Serdang, Selangor, Malaysia; 2 Department of Chemistry, Faculty of Science, University Putra Malaysia, 43400, UPM, Serdang, Selangor, Malaysia; 3 Department of Science, Faculty of Science, Technology and Human Development, University TunHussienOnn Malaysia (UTHM), 86400, Parit Raja, Batu Pahat, Malaysia; Southwest University, CHINA

## Abstract

A highly active and stable nano structured Pt/Mg_1-x_Ni_x_O catalysts was developed by a simple co-precipitation method. The obtained Pt/Mg_1-x_Ni_x_O catalyst exhibited cubic structure nanocatalyst with a size of 50–80 nm and realized CH_4_ and CO_2_ conversions as high as 98% at 900°C with excellent stability in the dry reforming of methane. The characterization of catalyst was performed using various kinds of analytical techniques including XRD, BET, XRF, TPR-H_2_, TGA, TEM, FESEM, FT-IR, and XPS analyses. Characterization of spent catalyst further confirms that Pt/Mg_1-x_Ni_x_O catalyst has high coke-resistance for dry reforming. Thus, the catalyst demonstrated in this study, offers a promising catalyst for resolving the dilemma between dispersion and reducibility of supported metal, as well as activity and stability during high temperature reactions.

## Introduction

In recent decades, the demand for alternative energy resources has steadily increased owing to the ongoing depletion of fossil fuels. Therefore, the utilization of greenhouse gases such as methane and carbon dioxide has received much attention. The reservation of methane is larger than crude oil because methane can be produced from various sources, including shale gas, fermented wastes, methane hydrates and others [1]. The syngas, consisting of hydrogen and carbon monoxide is an important feedstock for fuels and/or chemicals production in industry. The attractive feature of the dry reforming reaction is the utilization of CO_2_, which is a greenhouse effect gas. In general, the dry reforming reaction (Eq 1) is typically accompanied by the simultaneous occurrence of the reverse water–gas shift reaction (RWGS) (Eq 2).

$${\text{CH}}_{4}\;+\;{\text{CO}}_{2}\;\to 2{\text{H}}_{2}\;\Delta \text{H}=247\;\text{kJ}/\text{mol}$$ (1)

$${\text{CO}}_{2\;}+\;{\text{H}}_{2}\;\to \text{CO}\;+\;2H_{2}\text{O}\;\Delta \text{H}=41.1\;\text{kJ}/\text{mol}$$ (2)

A variety of catalysts have been developed for this reaction. Among them, the catalysts based on noble metals are reported to be less sensitive to coking than the nickel-based catalysts. However, poor stability caused by carbon deposition (Eq 3 and Eq 4) and aggregation of active Ni metal limits the industrial application of Ni catalysts in DRM reactions [2–3].

$${\text{CH}}_{4}\;\to {\text{C}}_{\left(\text{s}\right)}\;+\;2H_{2}\;\Delta \text{H}=+75\;\text{kJ}/\text{mol}$$ (3)

$$\text{CO}\to {\text{C}}_{\left(\text{s}\right)}\;+\;CO_{2}\;\Delta \text{H}=-171\;\text{kJ}/\text{mol}$$ (4)

It is well known that the performance of supported catalysts could be improved by selection of proper promoting materials. Supported transition metals, especially Ni, Ru, Rh, Pd, and Ir have considerable activity for reforming reactions. But supported nickel is preferred and more frequently used for industrial reforming processes because of its high availability and lower cost [4]. However, the Ni-based catalysts are readily deactivated by the deposition of carbon on the active centers.

Various promoters have been tested with metal-based catalysts in an attempt to decrease carbon deposition and prolong the life of the catalyst [5]. Maciel et al. [6] investigated the effect of metal-support interaction with the catalytic property of CuO/CeO_2_-TiO_2_ catalyst towards the conversion of hydrogen. However, the addition of ceria into the titania support hampers the transformation of anatase to rutile phase, facilitates the reduction of metal species and favors the metal-support interaction [7]. Choudhary et al. [8] reported CO_2_ reforming with simultaneous steam reforming or partial oxidation of methane to syngas over NdCoO_3_ perovskite-type mixed metal oxide catalyst and found that the methane-to-syngas conversion process occurs in an energy highly efficient manner, requiring little or no external energy. Li et al. [9] investigated the partial oxidation and steaming reforming of methane to produce syngas or hydrogen using Al_2_O_3_-coated SiC foam monolith catalyst. The study of Li et al. [9] revealed that the deposited carbon that originated from methane cracking in the upper part of the stainless steel reactor. Alternatively, Kim et al. [10] studied the effect between the catalytic reaction and electric discharge on methanol–steam reforming on Cu/ZnO/Al_2_O_3_ catalysts at various temperatures and discharge voltages. On the other hand, rhodium based catalyst shows considerable activity in dry reforming of methane process [11]. Recently, Ferreira-Aparicio et al. [12] have noticed that the addition of low Rh contents on Ni/γ-Al_2_O_3_ catalysts improves the activity and stability of catalysts making them more convenient for their use at industrial scale. Recently, a similar conclusion has been reached by Ocsachoque et al. [13] who studied on Rh–Ni/CeO_2_–Al_2_O_3_ catalysts for methane dry reforming. Even though, the rhodium based catalyst is a potential alternative with a small amount of carbon deposition and better catalytic performance, the dissociation of CO_2_ occurred at Rh site and thus the metallic Rh was oxidized to Rh^+3^ [12]. A way to improve catalytic stability and anti-coking performance, the low Pt content modified Mg_1-x_Ni_x_O catalyst studied for dry reforming of methane in this study. The effect of CH_4_ and CO_2_ concentration, catalyst concentration and temperature on conversion of syngas were studied. The comparison between the catalytic activity and stability as well as coke formation was also evaluated.

## Sample Preparation and Characterization

### Supports and Catalysts preparation

Mg_1-x_Ni_*x*_O (*x* = 0.00, 0.03, 0.07, 0.15) catalysts were prepared by the co-precipitation method as reported previously(Tomishige et al. 2004). Support MgO and promoter NiO was prepared using aqueous solution of Ni(NO_3_)_2_.6H_2_O (Merck; >99.0%) and Mg(NO_3_)_2_.6H_2_O (Merck; >99.0%). Potassium carbonate (K_2_CO_3_) (Merck; >99.7%) was used as a precipitant. After being filtered of the precipitant, the sample was washed with hot water. The sample was dried at 120°C for 12 hours and subsequently pre-calcined in air at 500°C for 5h. Subsequently, the sample was pressed into disks at 600 kg/m^2^, and then calcined at 1150°C for 20h.The preparation of catalyst was shown in Table 1. Finally, 1% Pt was impregnated using Pt(C_5_H_7_O_2_)_2_.H_2_O(Acros Chemicals; >99%) dissolved with dichloromethane. The catalysts were dried at 120°C after impregnation in air for 12 h.

**Table 1:** Preparation of catalyst.

Catalyst	Support (MgO) Mg(NO_3_)_2_.6H_2_O(g)	Promoter (NiO) Ni(NO_3_)_2_.6H_2_O (g)	Total weight of MgO and NiO (g)	Impregnation of the main catalyst (1% Pt) of Pt(acac)_2._ (g)
**Pt/MgO**	25.00	0.00	1	0.02
**Pt/Mg**_**0.97**_**Ni**_**0.03**_**O**	24.87	0.87	1	0.02
**Pt/Mg**_**0.93**_**Ni**_**0.07**_**O**	23.85	2.04	1	0.02
**Pt/Mg**_**0.85**_**Ni**_**0.15**_**O**	21.79	4.36	1	0.02

### Catalyst characterization

The thermo gravimetricanalysis (TGA) was carried out on a Mettler Toledo TG-DTAApparatus (Pt crucibles, Pt / Pt − Rh thermocouple) with the purge gas (nitrogen) flow rate of 30 ml min^−1^ and the heating rate of 10°C. min^−1^ from 50 to 1000°C.

X-ray diffraction analysis was performed using aShimadzudiffractometer model XRD 6000. The diffractometer employed Cu-Kα radiation to generate diffraction patterns from powder crystalline samples at ambient temperature. The Cu-Kα radiation was generated by Philips glass diffraction X-ray tube broad focus 2.7 kW type. The crystallite size D of the samples was calculated using the Debye–Scherrer's relationship^22^, Where D is the crystalline size, λ is the incident X-ray wavelength, β is the full width at half-maximum (FWHM), and θ is the diffraction angle.

The Fourier transform infrared (FT-IR) analysis was carried out with PerkinElmer spectrometer model 100 series (sample preparation UATR).

The total surface area of the catalysts was obtained using Brunauer–Emmett–Teller (BET) method with nitrogen adsorption at −196°C. Analysis was conducted using a Thermo Fisher Scientific S.P.A (model: Surfer Analyzer) nitrogen adsorption–desorption analyzer.

The Transmission Electron Microscopy (TEM, Model Hitachi H7100, Japan) was used to determine the crystal shape and Homogeneity of the Catalysts. Briefly, in deionized water, the powder was dispersed, dropped on to the Carbon-cover copper grids placed on a filter paper and at room temperature dried.

Field emission scanning electron microscopy (FE-SEM). The sample morphology was studied with JEOL Field Emission Scanning Electron Microscope (FE-SEM) model JSM 7600F at very high magnification by using field emission current. The particles of the samples were glued up on aluminum stud by using a double–sided tape. Then, it was coated with gold to make sure the better visibility of the surface and to prevent electrical charging of the sample during analysis.

The active site of the catalysts was evaluated by Temperature Programmed Reduction (TPR-H_2_) using hydrogen was conductedby Thermo Finnegan TPDRO 1100 apparatus Equipped with a thermal conductivity detector. In the reactor, about 0.05 g of catalyst was placed and treated under 150°C for 40 min in N_2_ (20 ml/min). The analysis of Hydrogen 5.51% in Argon was carried out between 50°C and 950°C under argon flow (10°C min^−1^, 25 ml min^−1^)and detected by a thermal conductivity detector.

XPS spectra were obtained using Kratos Axis Ultra DLD system, equipped with a monochromatic Al Kα (1486.6 eV), dual x-ray sources (Al & Mg), an argon etching system for sample cleaning and depth profiling, parallel imaging XPS, AES, ISS and Vision software for controlling the system. The base pressure of the analyzer chamber was 1 × 10^−10^Torr. The excitation sources, X-ray gun was operated at a combination of 20 mA of emissions current and 15 kV voltages. The hemispherical analyzer was operated in the fixed analyzer transmission (FAT) mode for both wide and narrow scanning. This value is set at 100 eV and 40 eV of pass energy respectively. The region of interest for the narrow scan is corresponding to Mg2p, Ni2p, Pt4f, and O1s photoelectron signal. The carbon charging correction refers to the binding energy of adventitious carbon at the binding energy of 285 eV. This highly sophisticated equipment is considered as a non-destructive analysis technique due to soft x-ray production to induce photoelectron emission from the sample surface. Therefore, the equipment would provide information about surface layers or thin film structures (about the top 10–100 Å of the sample).

### Catalyst evaluation

**Fig 1:**
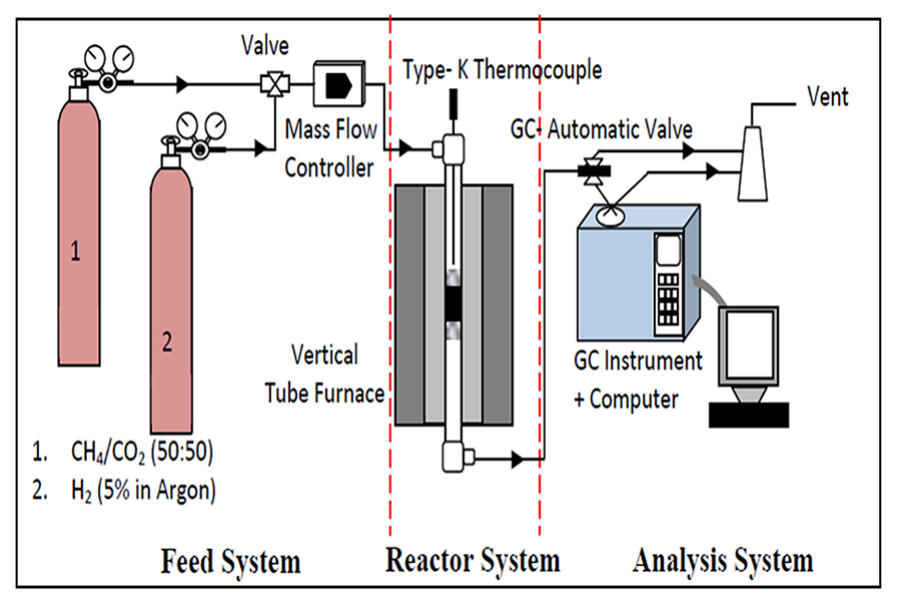
Experimental system for dry reforming of methane.

The catalytic evaluation for dry reforming of methane with CO_2_ (DRM) towards syngas (H_2_/CO) production was carried out using a fixed bed stainless steel micro-reactor (i.d. Ø = 6 mm, *h* = 34 cm) as shown in Fig 1. The reactor was connected to a mass flow gas controller (SIERRA instrument) and an online gas chromatography (GC) (Agilent 6890N; G 1540N) equipped with varian capillary columns HP-PLOT/Q and HP-MOLSIV. Prior to reaction, approximately 0.02 g catalyst was reduced by flowing 5% H_2_/Ar (30 ml min^-1^) at 700°C and holding for 3h. The reforming reaction was performed by flowing the feed, a gas mixture consisting of CH_4_/CO_2_ in (2/1) and (1/1) mol and CH_4_/CO_2_/O_2_ (53.75/45/1.25), at a rate of 30 ml. min^-1^, the reforming has been studied from 700 to 900°C at 1 atm, then holding for 10h (1 atm, GHSV = 15000 ml h^-1^ g^-1^cat).

The reduction step was aimed to reduce (Pt^2+^) phase to Pt metal (Pt^o^) phase. The catalyst tested was placed in the middle of reactor vertically and held in place by plugs of quartz wool. In order to control and ensure the reaction temperature, a thermocouple was placed into the catalyst chamber. The selectivity of the CO was determined by ratio of moles of CO produced to sum of the moles of methane and CO_2_ converted. The conversion of CH_4_, CO_2_, H_2_ and CO were calculated according to the following equations (Eqs 5–9).

$$CH_{4}\;conversion\;\%=\frac{CH_{4\;in}-CH_{4\;out}}{CH_{4\;in}}*\;100\;(\%)$$ (5)

$$CO_{2}\;conversion\;\%=\frac{CO_{2\;in}-CO_{2\;out}}{CO_{2\;in}}*\;100\;(\%)$$ (6)

$$H_{2}\;selectivity\;\%=\frac{H_{2}}{2\left[CH_{4\;in-}\;CH_{4\;out}\right]}*\;100\;(\%)$$ (7)

$$CO\;selectivity\;\%=\frac{CO}{\left[CH_{4\;in-}\;CH_{4\;out}\right]+\left[CO_{2\;in}-CO_{2\;out}\right]}*\;100\;(\%)$$ (8)

$$\frac{H_{2}}{CO}ratio=\;\frac{H_{2}\;selectivity\;\%}{CO\;selectivity\;\%}$$ (9)

## Results and Discussion

### Characterization of catalysts

**Fig 2:**
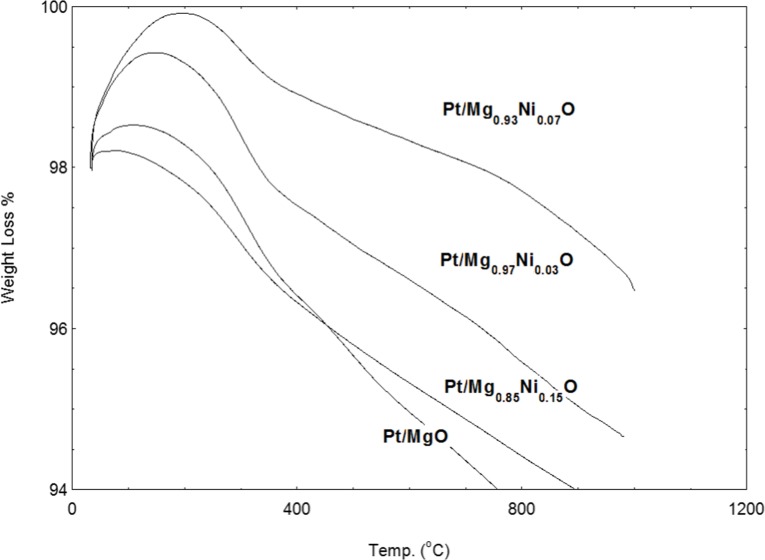
TG of the catalysts (a) Pt/MgO (b) Pt/Mg_0.85_Ni_0.15_O(c) Pt/Mg_0.97_Ni_0.03_O (d) Pt/Mg_0.93_Ni_0.07_O.

The TGA analysis of the prepared catalysts was illustrated in Fig 2. The weight loss of 4% observed at temperatures 41°C and 266°C could be attributed to the removal of moisture from the catalyst, as shown in Fig 2A. The compound was found to be thermally stable at 500°C which could be associated with the high melting point of MgO (2852°C) and NiO (1955°C). The similar natures of thermal decomposition of other catalysts were observed in Fig 2B–2D.This observation is in agreement with the results reported in the literatures [14–15].

**Fig 3:**
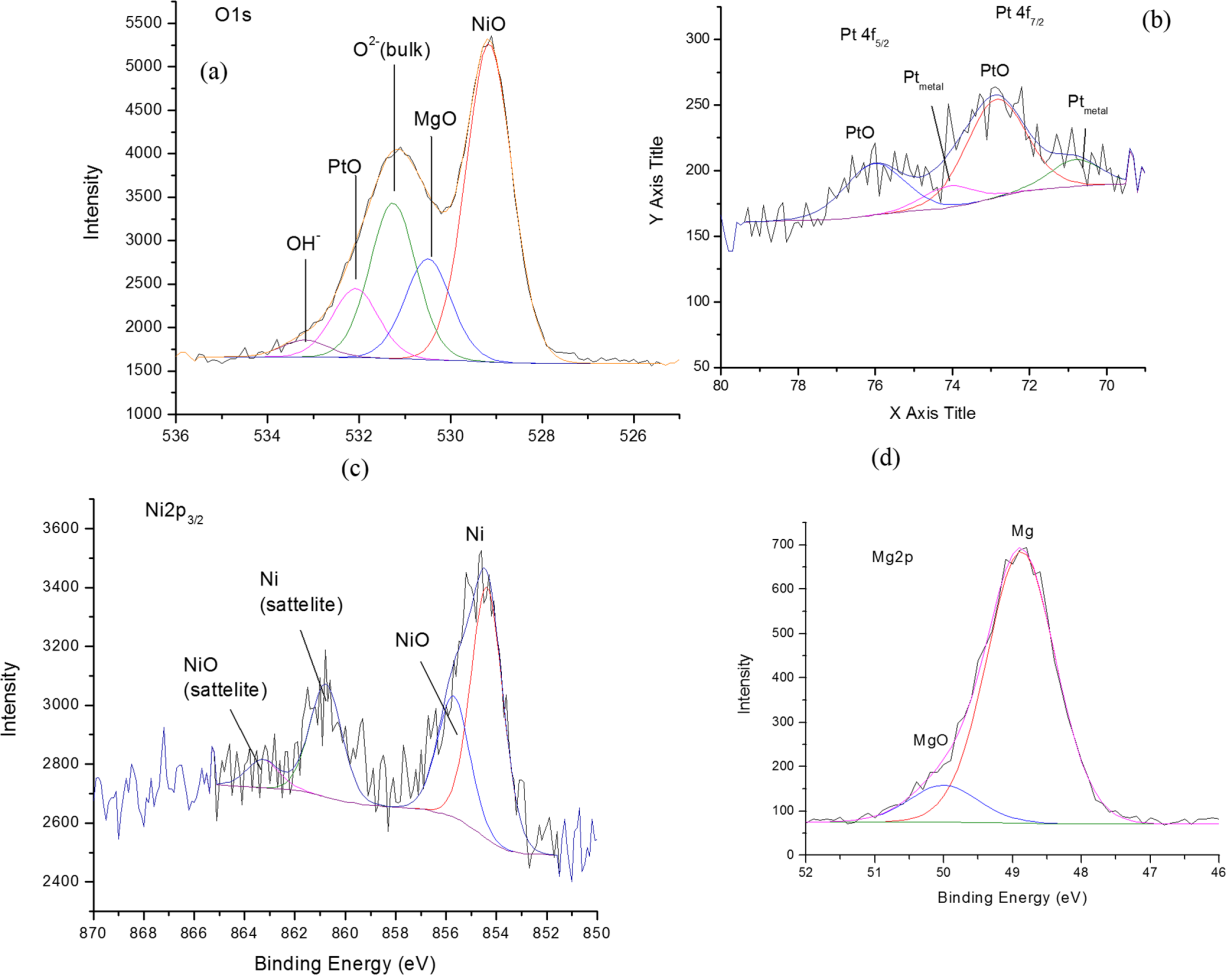
XPS narrow scan of the reduced catalyst. (a) O1s (b) Pt4f (c) Ni2p (d) Mg2p.

Information on the surface composition of the catalyst was obtained by XPS analysis. Surface study of the catalyst at a few nanometer layer 3–12 nm of its uppermost using XPS indicate that there is a photoelectron signal from O1s, Mg2p, Ni2p and Pt4f, as illustrated in Fig 3. A deconvolution of a O1s photoelectron signal shows that it is contributed by five types of oxygen species which is contributed by O^2-^ (from the bulk), Mg-O, Ni-O, Pt-O and hydroxyl (OH^-^) component. This is also a significant splitting of this O1s spectrum, whereby Ni-O exhibit as the highest intensity of photoelectron signal in the lower binding energy region compared to the others. However, the O^2-^, Ni-O, Pt-O and OH^-^ were detected under a similar photoelectron envelope. The contribution of the OH^-^ component is considered very low at the binding energy of 533.0 eV. On the other hand, the narrow scan of Mg2p, Ni2p and Pt4f revealed that the oxide species of these metals are a mixture of Mg-O and Mg(OH)_2_, Ni and Ni-O, and Pt-O and Pt respectively. The existence of two components of hydroxyl species by Ni and Mg indicated that these metals are easy to react with water vapor compound (H_2_O) [16–17].

**Fig 4:**
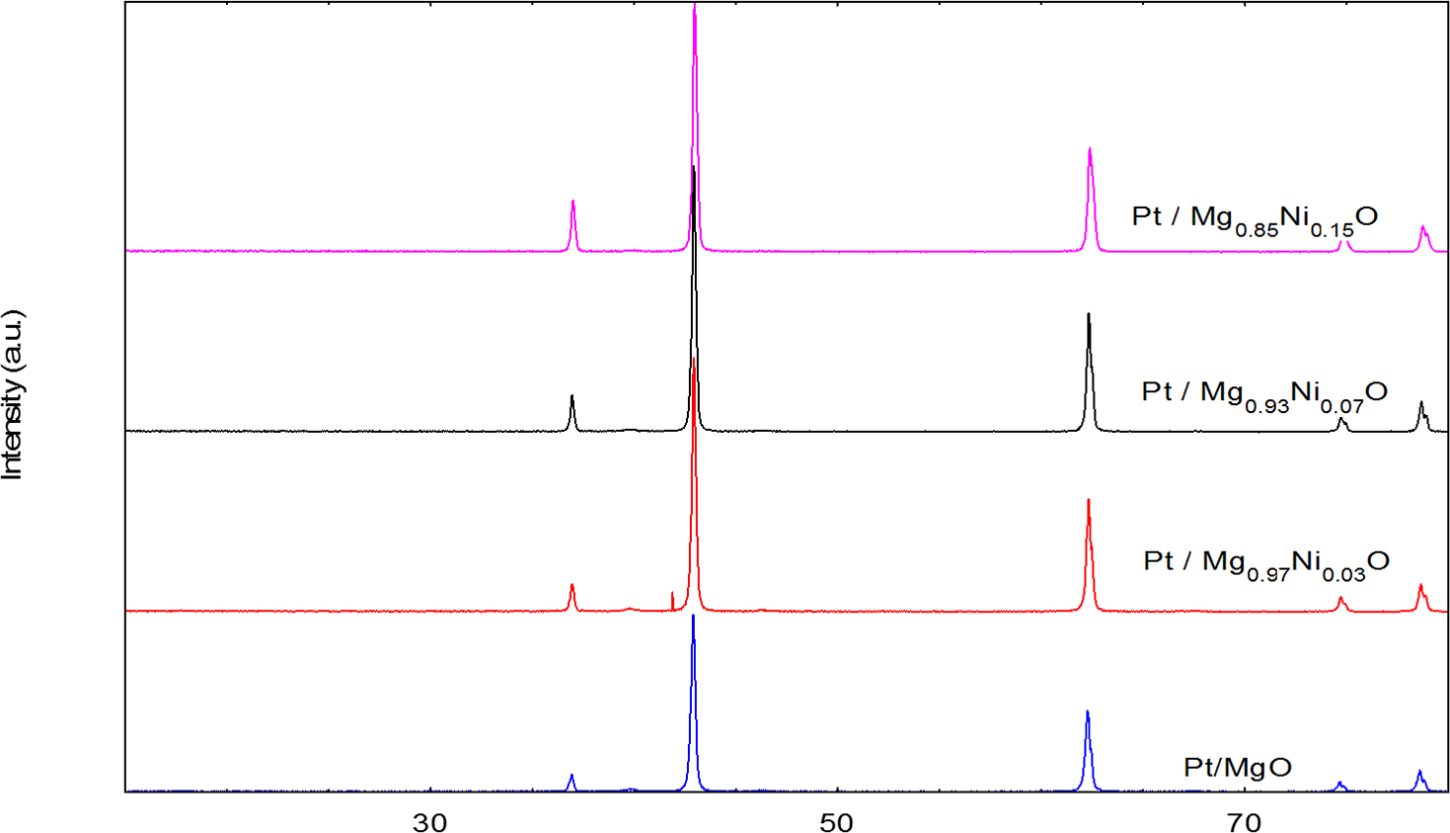
XRD results of catalysts (a) Pt/MgO(b) Pt/Mg_0.97_Ni_0.0_ (c) Pt/Mg_0.93_Ni_0.07_O (d) Pt/Mg_0.85_Ni_0.15_O.

X-Ray Diffraction (XRD) patterns of the catalysts are shown in Fig 4. The diffraction peaks at 2θ = 39.7, 42.9, 62.5, 75.0 and 79.0° were associated with the cubic form of magnesia (JCPDS file no.: 00-024-0712). The value of 2θ at 37.3, 43.3, 62.7, 75.1, and 79.8 were corresponds to the cubic form of NiO (JCPDS file no.: 00-001-0800). No diffraction peak of Pt in the patterns was observed as it exists in very low concentration. This observation agrees well with the results reported by Grange [18]. The average crystalline size was determined by the diffraction peak of the Mg plane in XRD patterns using the Debye-Scherrer equation (Table 2). The size of the crystals were 44.7, 42.4, 40.4 and 38.7 nm for Pt / Mg_0.97_Ni_0.03_O, Pt/ Mg_0.93_Ni_0.07_O, Pt / Mg_0.85_Ni_0.15_O and Pt/MgO, respectively. The results revealed that the crystal size was decreased with increasing Ni concentration. This could be due the effect of platinum that remain bounded on the surface of the samples and inhibit the growth of magnesia crystallites [19]. Further, it was reported by Jogalekar et al. [20] that the addition of nickel oxide coluld prevent agglomeration of the magnesia particles and thus, decrease in particle size.

**Table 2:** The main textural properties of fresh catalysts.

Sample name	Specific ^a^Surface Aream^2^/g	Pore Volume Cm^3^/g	Pore volume to S_BET_ ratio 10^-9^m	Pore radius °A	Pt ^b^Loading Wt %	Average ^c^Crystal size nm
Pt / Mg_0.97_Ni_0.03_O	11.64	0.17	17.6	18.25	0.98	44.7
Pt / Mg_0.93_Ni_0.07_O	6.72	0.06	15.7	18.26	0.94	42.4
Pt / Mg_0.85_Ni_0.15_O	5.44	0.04	10.6	18.24	0.93	40.4
Pt / MgO	10.46	0.41	48.4	18.26	0.95	38.7

a. Specific surface area calculated by BET method.

b. Determined by the XRF method.

c. Determined by the Debye-Scherrer equation of the Mg (200) plane of XRD.

The crystal system for all the samples was cubic, as confirmed by TEM and FESEM analyses.

**Fig 5:**
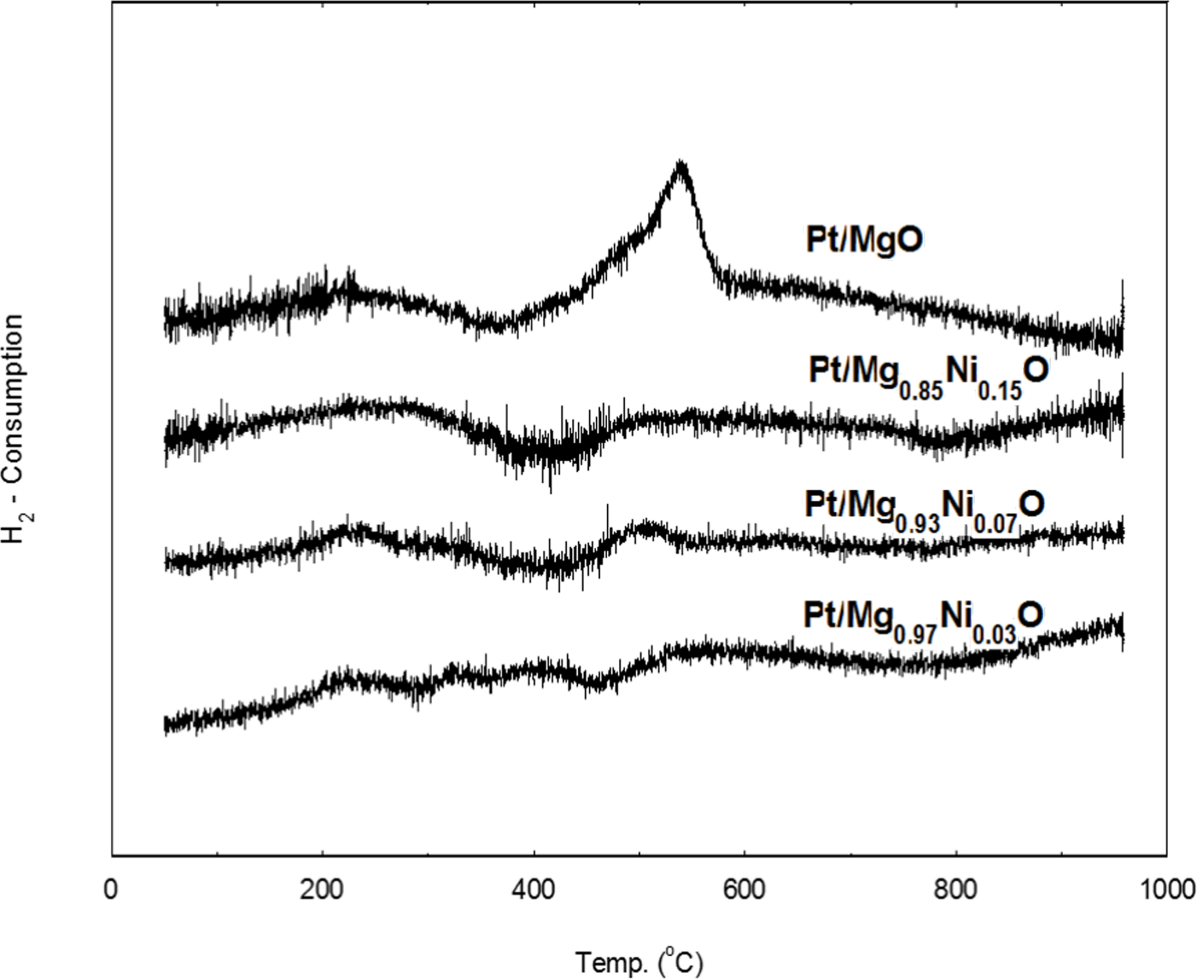
H_2_-TPR profiles o catalysts (reduced in a 5% H_2_/Ar stream at a temperature ramp of 10°C/min). (a) Pt/Mg_0.97_Ni_0.03_O (b) Pt/Mg_0.93_Ni_0.07_O (c) Pt/Mg_0.85_Ni_0.15_O (d) Pt/MgO.

The H_2_-TPR has been extensively used to characterize the Ni reducibility of the reforming Ni catalysts. The TPR-H_2_ profiles for the Pt/Mg_1-x_Ni_x_O catalysts are presented in Fig 5. For comparison the reducibility and different Ni concentration in the MgO support platinum catalysts were investigated. Fig 5 shows all of the catalysts had a broad and overlapping H_2_ consumption peak at 50 to 950°C, indicating the different interactions of Pt with the support [21]. The peaks shown in TPR profile (Fig 5) at 215, 220 and 219°C could be attributed to the reduction of Pt-O to Pt. The other broad peaks at 465, 470 and 485°Cfor Pt/Mg_0.97_Ni_0.03_O, Pt/Mg_0.93_Ni_0.07_O, and Pt/Mg_0.85_Ni_0.15_O catalyst were associated with the reduction of Ni-O to Ni^0^. The reduction peak of NiO is shifted to a lower temperature when it is deposited on the MgO support [22]. It is likely that the better-dispersed Ni and an appropriate interaction between metal and support increase the Ni reducibility. The TPR-H_2_ profiles confirmed that 700°C was the appropriate re-reduction temperature for the complete reduction of catalysts. These peaks might relate to the reduction of nickel oxide particles supported on the outside surface and inside the porous structure of the catalysts, which can be clearly distinguished by TEM results.

**Fig 6:**
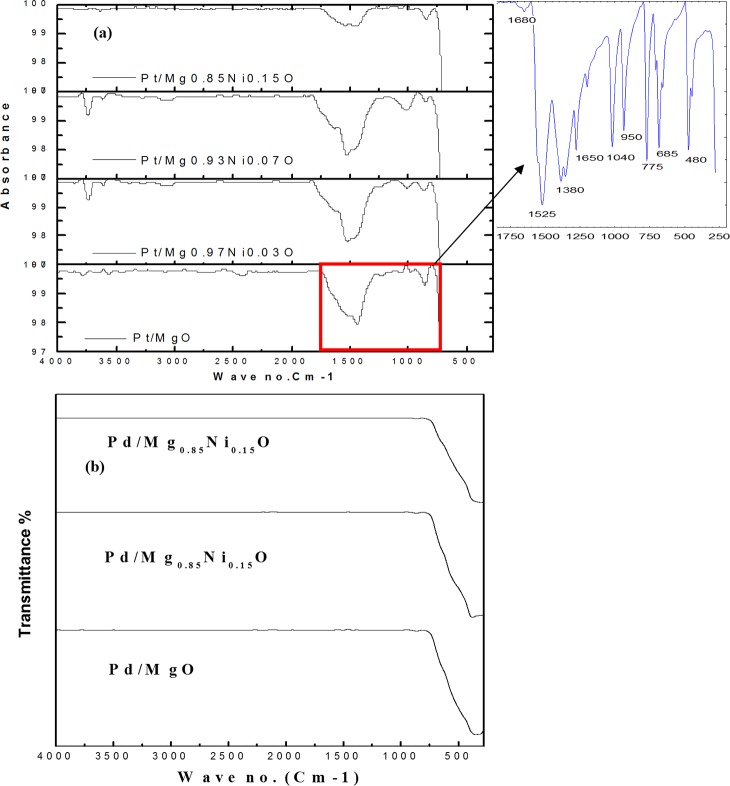
FT-IR of (a) unreduced and (b) reduced catalysts.

FTIR spectra for un-reduced and reduced catalysts depicted in Fig 6. As shown in Fig 6A, the sharp peaks around 1525 Cm^-1^ which represents the C = C stretch for each catalyst. Broad peaks at 3050 Cm^-1^ represented the C-H bond. Moreover, the band at 1680 Cm^-1^ can be assigned to the vibration of carbonyl (C = O) group in the acetylactonate on the catalyst. The band at 1380 cm^-1^ and 1040 cm^-1^ can be assigned to the existence of C-H bending C-O stretch, respectively. The band in the region 680 to 700 Cm^-1^ was attributed to the NiO stretching, vibration mode, whereas, the vibration bonds for Pt-O and MgO exists in the far IR region [23, 15]. However, all the acetylactonate peaks was disappearing after reduction at 700 °C, as shown in Fig 6B.

The BET specific surface area (S_*BET*_) value and pore properties of catalyst supports are shown in Table 2. After impregnation, the S_*BET*_ value and pore volume decreased in all three of the catalysts. This phenomenon might be caused by pore blocking during the impregnation process. Consistent with earlier reports [24], the platinum doped Mg_0.97_Ni_0.03_O sample showed a higher surface area as compared to the magnesium oxide sample. The dispersed platinum particles along with the support prevent the agglomeration of catalyst particles leading to an enhanced surface area. Furthermore, the pore volume of the samples was decreased trend with increasing nickel oxide in the magnesium oxide. It can be assumed that the incorporation of NiO prevents surface area loss during high temperature calcinations resulting in increase the surface area of the samples. There was no obvious connection between the S_*BET*_ value and pore volume of catalysts, but the pore volume/S_*BET*_ratio decreased in the order Pt / MgO > Pt / Mg_0.97_Ni_0.03_O > Pt / Mg_0.93_Ni_0.07_O > Pt / Mg_0.85_Ni_0.15_O, which was in accordance with the NiO dispersion order. This result is consistent with the result reported by Saha et al. [24] that the high pore volume/S_*BET*_ratio contributes to high catalytic performance. The XRF results in Table 2 show that the Pt-loading was slightly lower than the set value of 10%. This might be caused by weight loss during the pre-calcinations of the supports, resulting in a higher Ni content in the catalysts [25].

**Fig 7:**
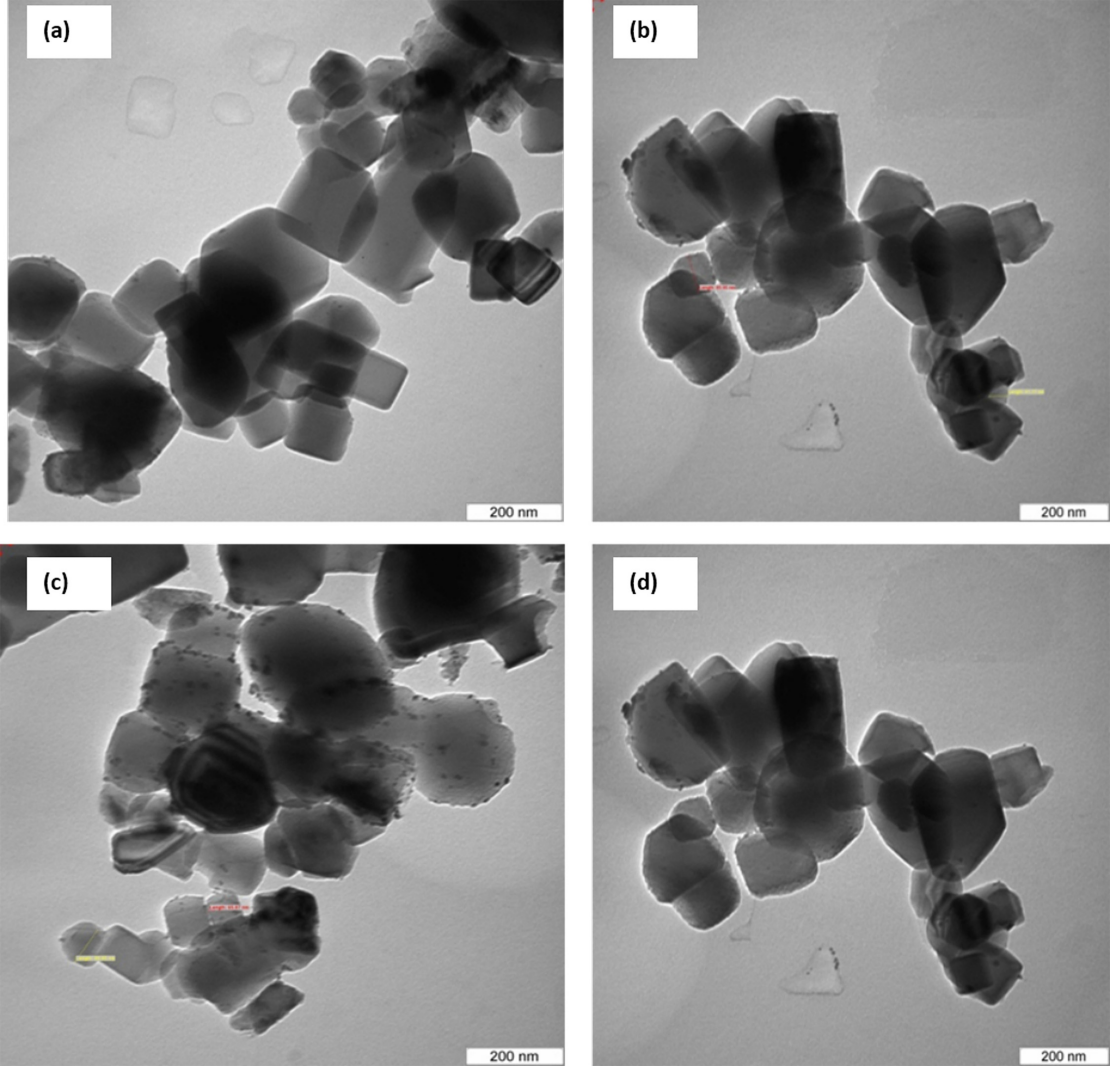
TEM image of catalysts(a) Pt/MgO(b) Pt/Mg_0.97_Ni_0.03_O(c) Pt/Mg_0.93_Ni_0.07_O (d) Pt/Mg_0.85_Ni_0.15_O.

**Fig 8:**
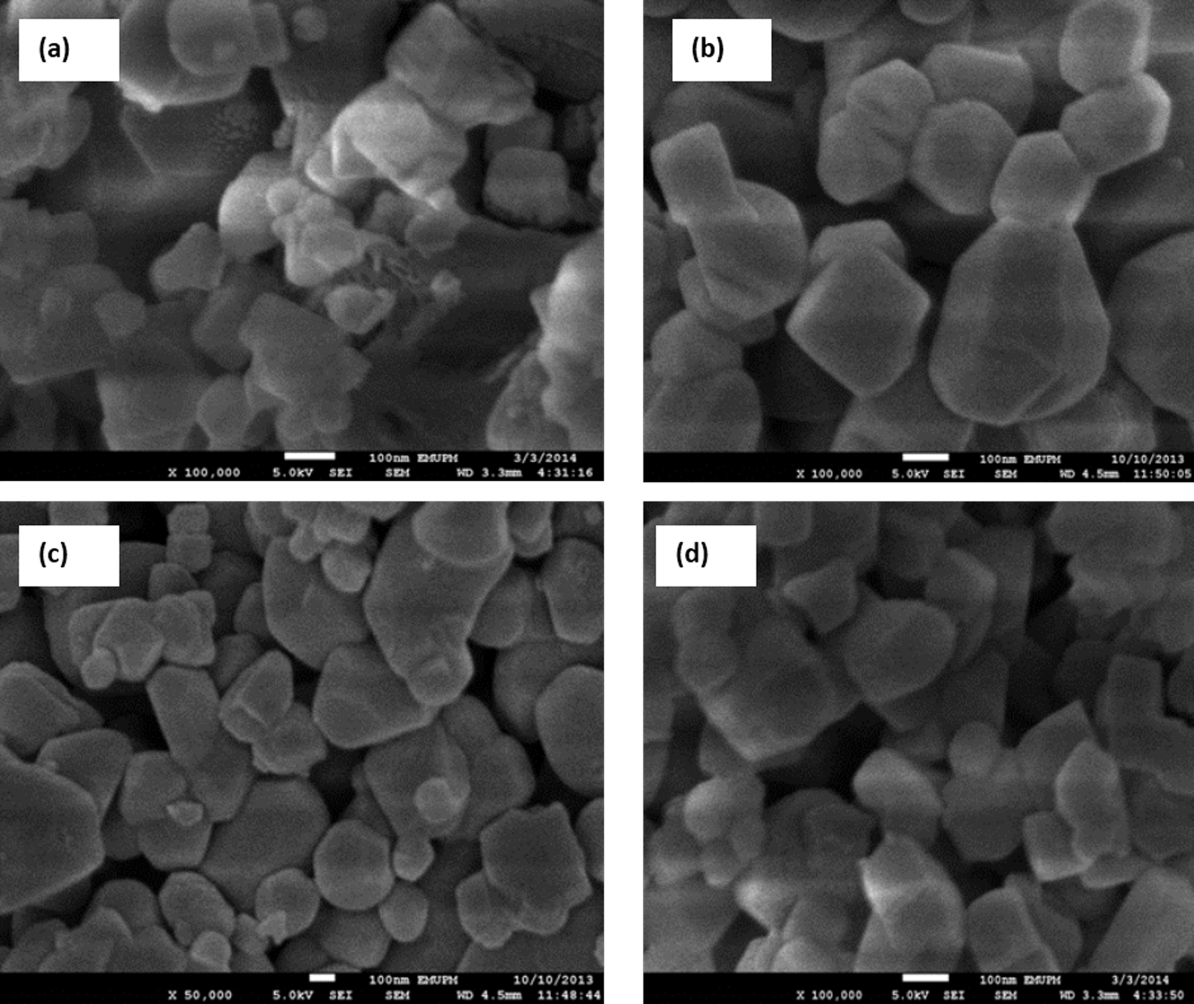
FESEM image of catalysts (a) Pt/MgO(b) Pt/Mg_0.97_Ni_0.03_O, (c) Pt/Mg_0.93_Ni_0.07_O (d) Pt/Mg_0.85_Ni_0.15_O.

To estimate the morphology of the catalyst, TEM images of one samples were acquired. Fig 7 illustrates the morphology and size distribution of the synthesized catalysts. The smallest crystals were characterized by TEM, which reveals crystals with a size of about 50–80 nm, and even resolves the cubic structure. The average size of the Pt nanoparticle with various sizes from 4–7 nm. The morphology of the catalyst could be supported by FESEM analysis (Fig 8). The supports particles are regular inshape Pt particles were uniformly distributed on supports. A two-dimensional cubic texture is assigned to the catalyst and uniform pore size of ~5 nm was observed (Table 2). This is consistent with the results reported by other researchers [26].The particle size of supported Pt increased in the order of Pt/Mg_0.85_Ni_0.15_O <Pt/Mg_0.93_Ni_0.07_O <Pt/Mg_0.97_Ni_0.03_O, which was in agreement with the Scherrer’s equation results.

### Catalytic performance

#### Effect of reactant concentration on conversion

**Fig 9:**
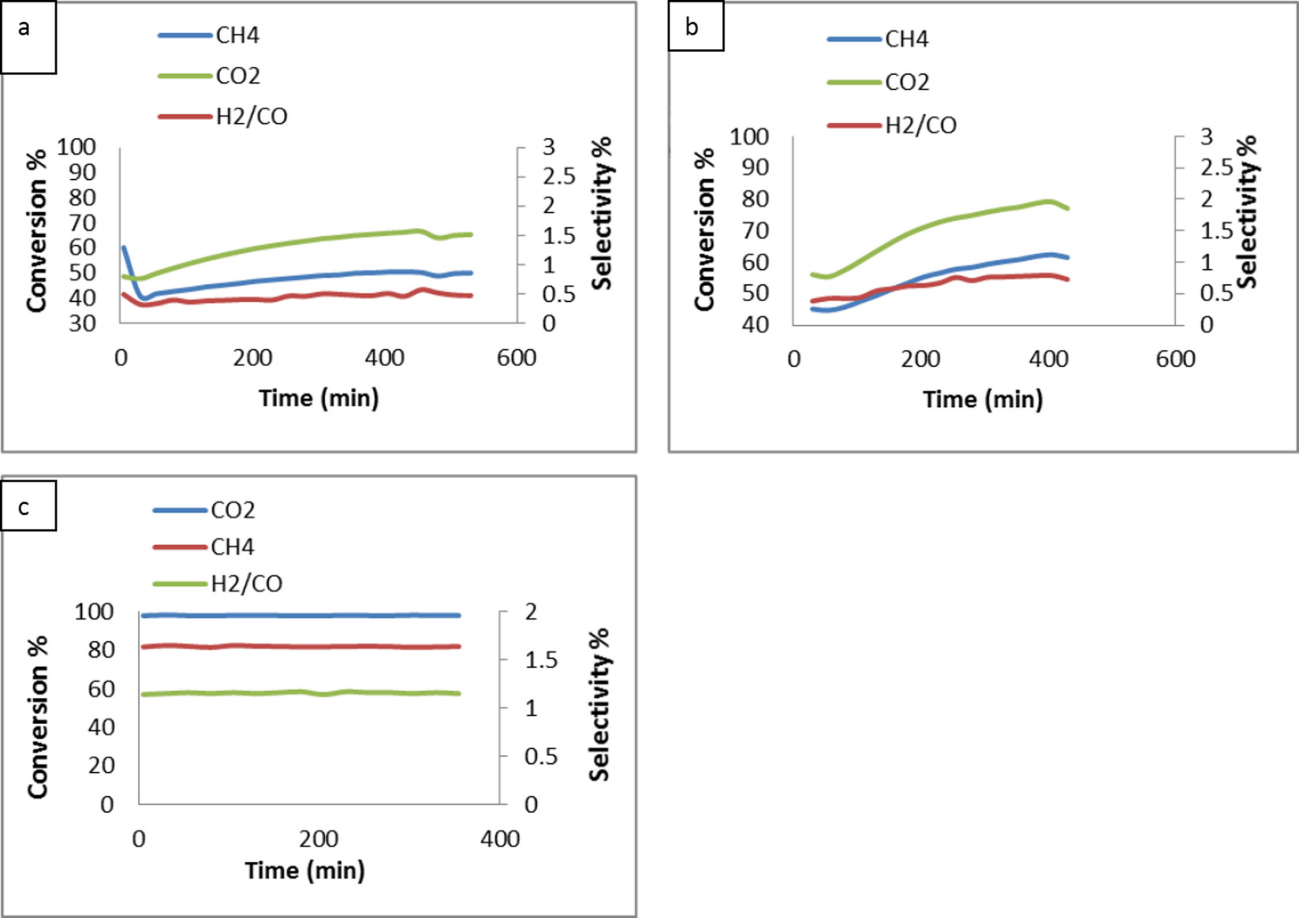
Effect of catalyst type on CH_4_, CO_2_ and H_2_/CO conversion with CO_2_/CH_4_ = 1:2 at 900°C; (a) Support(MgO); b) Support with promoter (Mg_0.85_Ni_0.15_O) and c) Catalyst (Pt/Mg_0.85_Ni_0.15_O)

**Fig 10:**
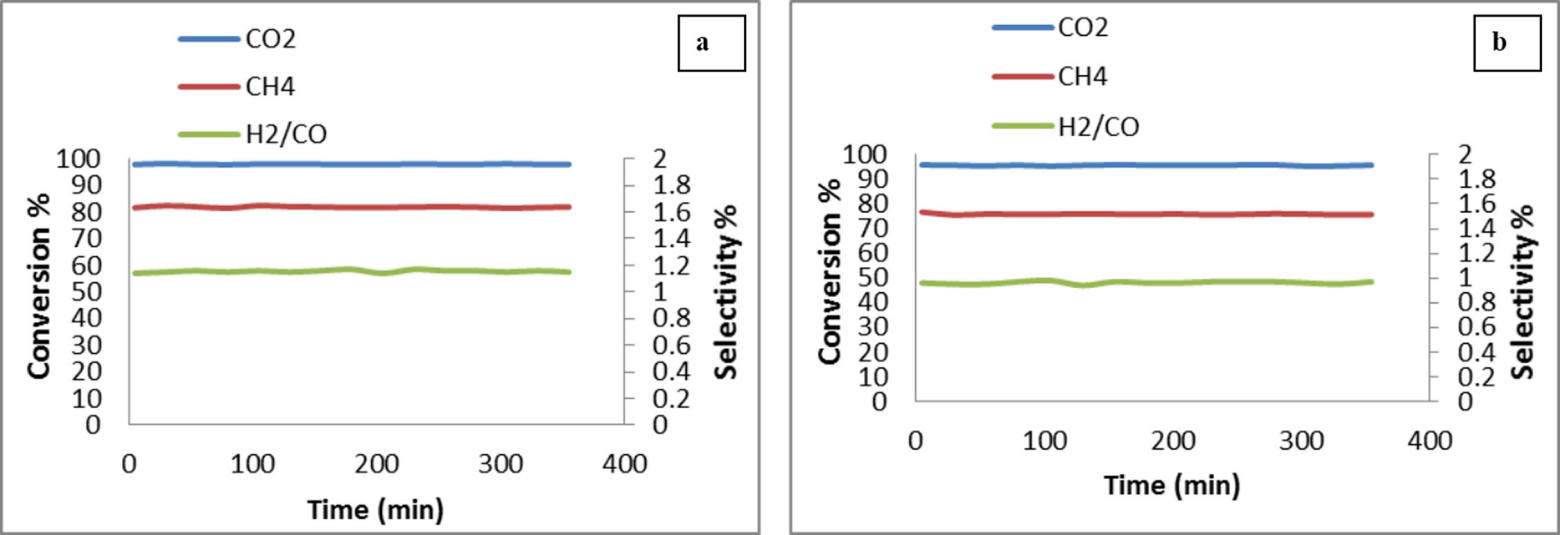
Effect of CH_4_/CO_2_ ratio on the on CH_4_, CO_2_ and H_2_/CO conversion at 900°C; (a) 2:1; b) 1:1.

Effect of catalyst type on CH_4_, CO_2_ and H_2_/CO conversion with the CO_2_/CH_4_ ratio of 1:2 at 900°C was shown in Fig 9. The conversion of CH_4_ and CO_2_ to syngas was expressed by the H_2_/CO ratio. The detection of H_2_ and CO after the blank tests (reaction without catalyst) performed at 900°C was indicated the decomposition of methane reaction according to the reaction (Eq 1). When MgO was used alone as a support without NiO, the conversion of CH_4_ and CO_2_ was low, as shown in Fig 9A. These results indicated that the conversion of syngas using MgO is very low. When support with promoter (Mg_0.85_Ni_0.15_O) was used, the conversion of syngas was increased (Fig 9B). The enhancement was due to the presence of promoter NiO. On the other hand, the conversion and the H_2_/CO ratio increased sharply for the case of Pt/Mg_0.85_Ni_0.15_O catalyst (Fig 9C). The result revealed that the main catalyst (Pt metal) doped on the support plays the main role in the catalyst. By using the Pt/Mg_0.85_Ni_0.15_O catalyst, the average conversion of CH_4_ and CO_2_ gases at the ratio (2:1) was 81 and 97%, respectively, whereas the conversion of the gases at the ratio (1:1) was 76 and 95% respectively (Fig 10). This could be due the best resistance to the deactivation of the catalyst and thus, the high selectivity towards the conversion of H_2_ and CO was observed.

#### Effect of catalyst concentration on conversion

**Fig 11:**
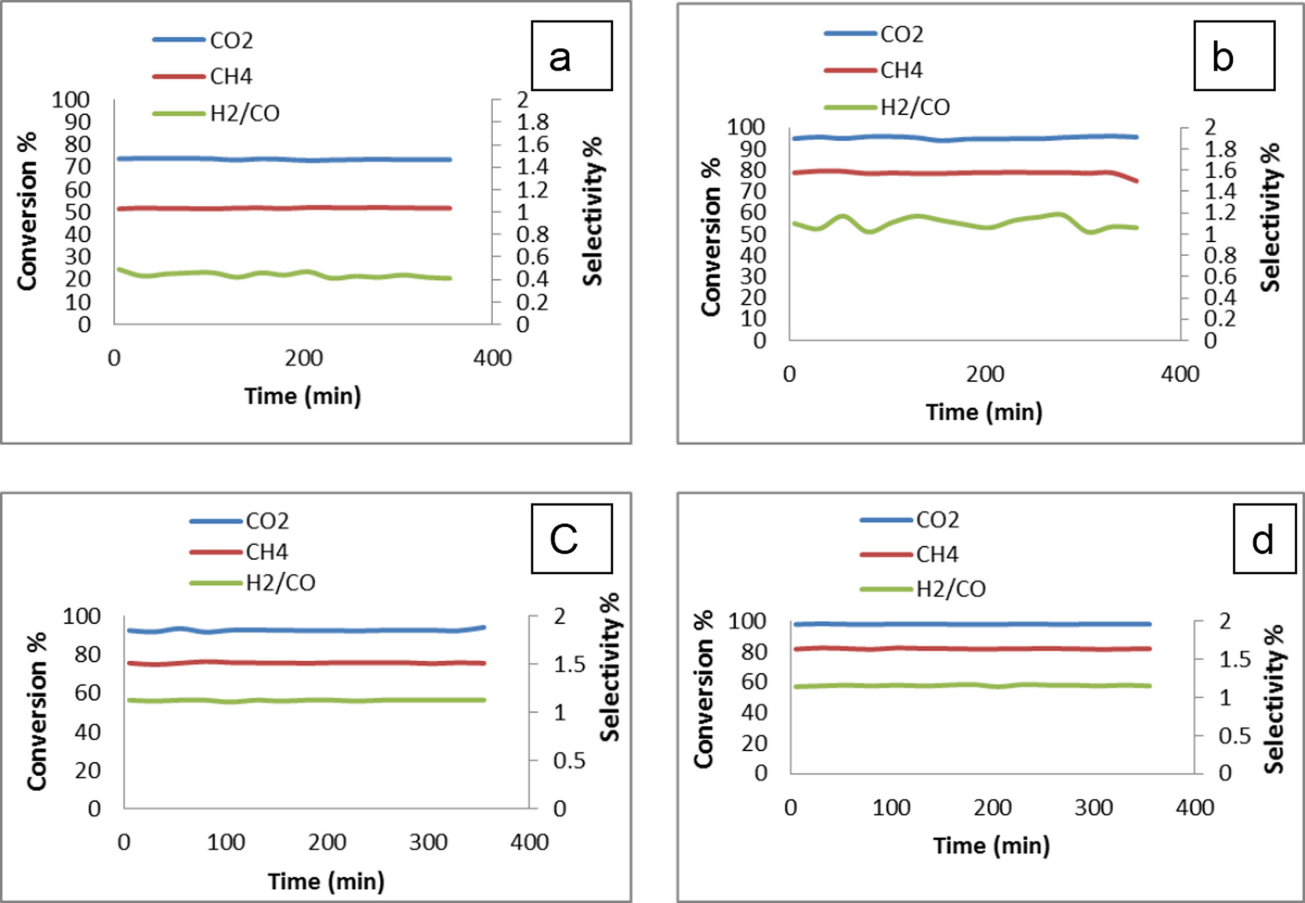
Effect of catalyst concentration on H_4_, CO_2_ and H_2_/CO conversion with CO_2_/CH_4_ = 1:2, at 900°C; (a) Pt/MgO b) Pt/Mg_0.97_Ni_0.03_O c) Pt/Mg_0.93_Ni_0.07_O d) Pt/Mg_0.85_Ni_0.15_

The effect of catalyst concentration on conversion was shown in Fig 11. The increasing order of conversion of CH_4_, CO_2_ and the H_2_/CO ratio was Pt/MgO <Pt/Mg_0.97_Ni_0.03_O < Pt/Mg_0.93_Ni_0.07_O < Pt/Mg_0.85_Ni_0.15_O. No change in conversion, selectivity of CH_4_ and CO_2_ with increasing the Ni concentration in the support,was observed. This could be attributed to the formation of strong Lewis basicity with a metal oxide support. The increase of the Lewis basicity of the support could be enhanced the ability of the catalyst to chemisorbs CO_2_ in the CO_2_ reforming of methane.The adsorbed CO_2_ can reacts with C to form CO, resulting the reduction of coke formation according to the equation (Eq 10).

$${\text{CO}}_{2}\;+\;\text{C}\;\to 2\text{CO}\;\Delta \text{H}=171\;\text{kJ}/\text{mol}$$ (10)

The formation of NiO–MgO solid solution provides a unique approach to inhibit the carbon deposition. The support MgO is strong Lewis bases, which has a strong adsorption of CO_2_ to reduce or inhibit carbon deposition. Furthermore, XPS results revealed that the reduction of NiO in NiO–MgO solid solution was much more difficult than that of pure NiO, leading to small nickel metal particles formed on the surface [27–28].

#### Effect of temperature on conversion

**Fig 12:**
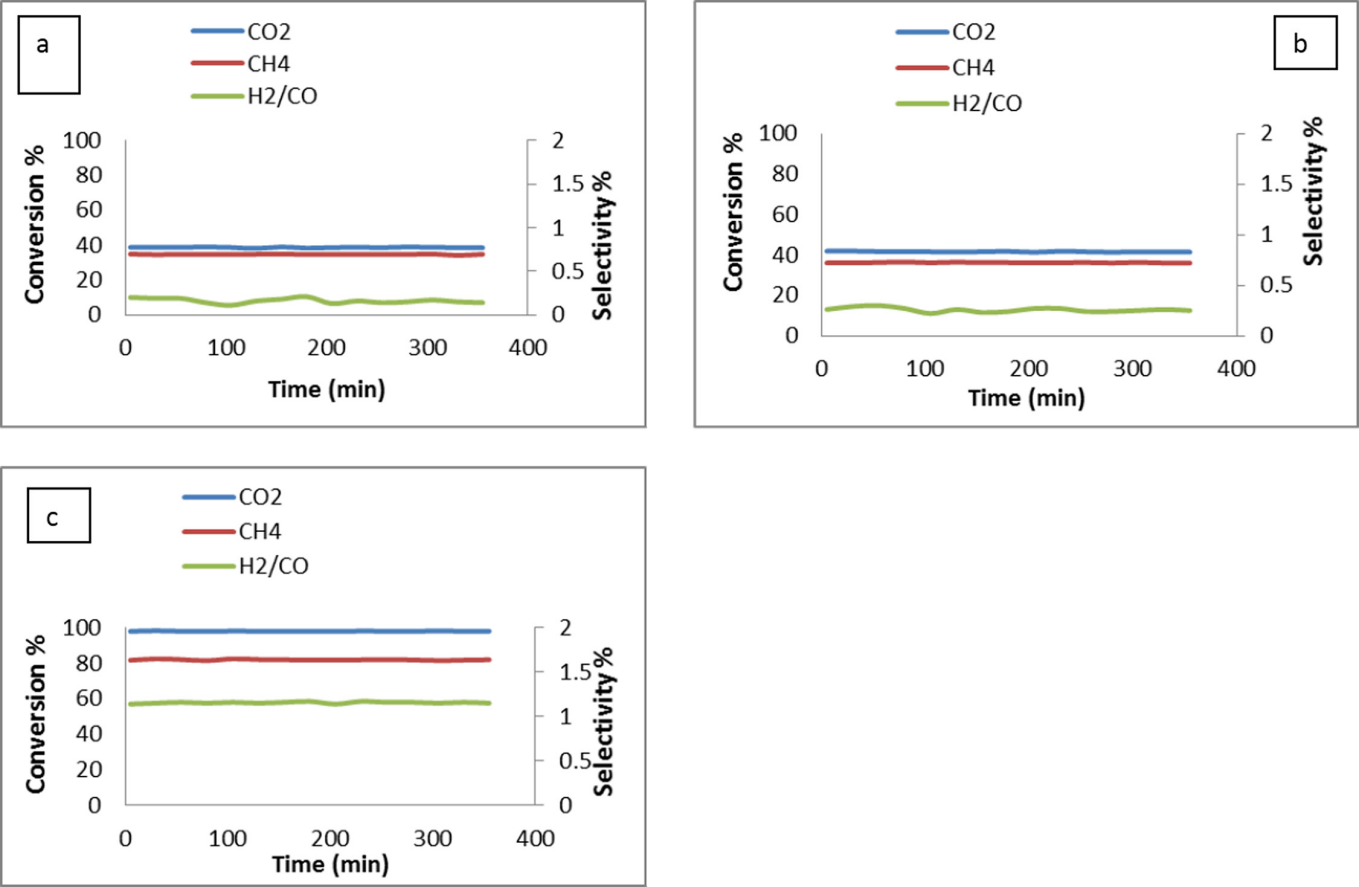
Effect of temperature on H_4_, CO_2_ and H_2_/CO conversion for Pt/Mg_0.85_Ni_0.15_O catalyst with CO_2_/CH_4_ = 1:2; (a) 700°C (b)800°C (c) 900°C

Fig 12 shows the activity and selectivity results of the Pt/Mg_0.85_Ni_0.15_O catalyst. The conversion of both CH_4_:CO_2_ (2:1) increased as the temperature was increased from 700 to 900°C. The dry-reforming of methane reaction is a strong endothermic reaction (Eq 1) and resulting the high temperature can be increased the conversion rate, as observed in earlier studies [29]. The CH_4_ conversion on Pt / Mg_0.85_Ni_0.15_O increased from 34% to 82% and the CO_2_ conversion increased from 38% to 98% when the temperature increased from 700 to 900°C. At temperatures above 900°C, no increase in CH_4_ and CO_2_ conversion was evident. The H_2_/CO ratio of the catalyst at various temperatures is shown in Fig 12A–12C. The H_2_/CO ratio of the samples was less than 1 at the temperature above 900°C. The reverse water-gas-shift reaction (Eq 11) can consume the additional H_2_ and produces CO, which lowers the H_2_/CO ratio [29].

$${\text{CO}}_{2}\;+\;{\text{H}}_{2}\to \text{CO}\;+\;{\text{H}}_{2}\text{O}\;\Delta \text{H}=\;+41.2\;\text{kJ}/\text{mol}$$ (11)

### Stability tests

**Fig 13:**
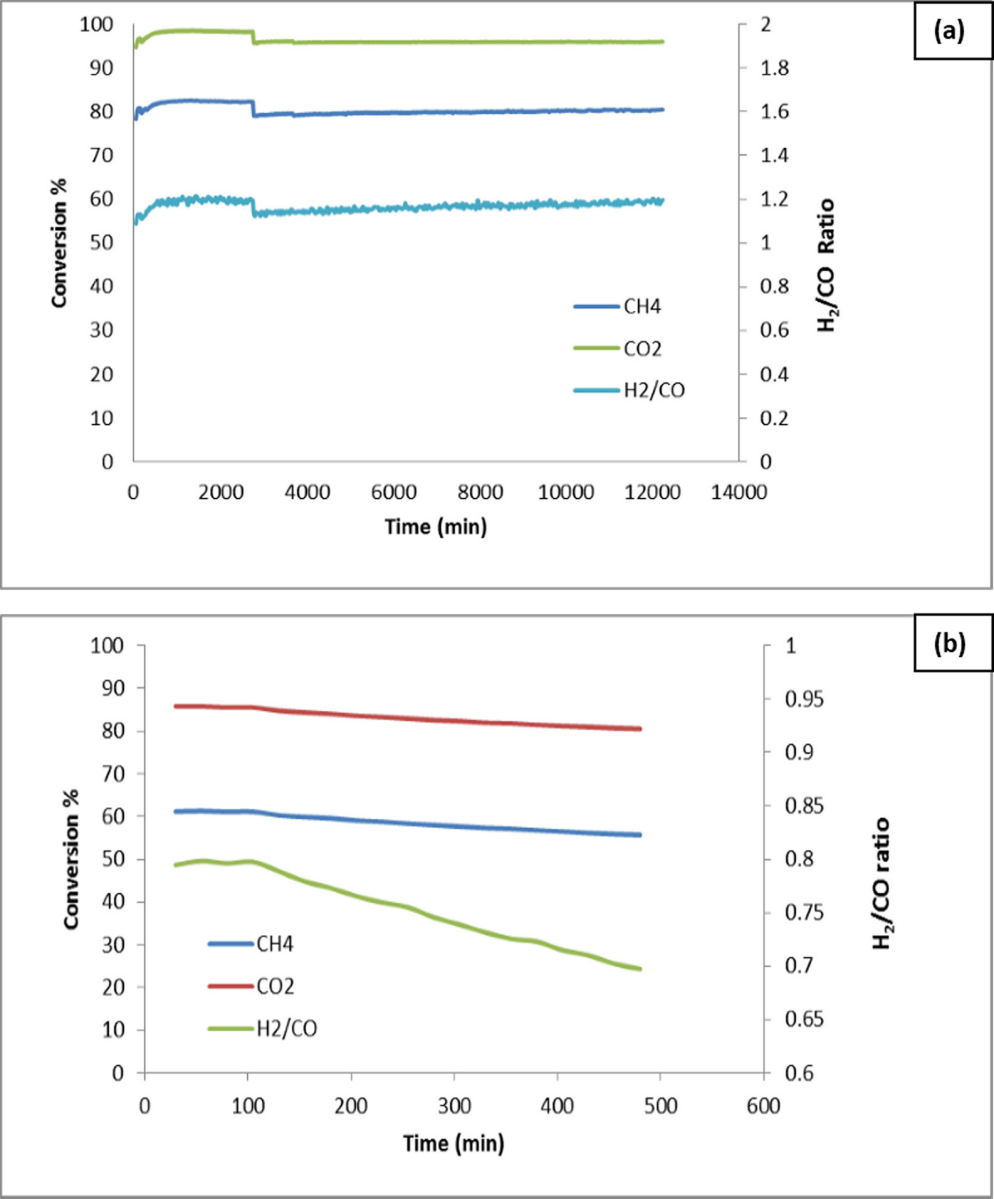
Stability tests of Pt / Mg_0.85_Ni_0.15_O fresh catalysts (a) and recycled catalysts at 900°C for 200 h (GHSV = 15000 ml cat^-1^h^-1^, atmospheric pressure).

Temperature tests, as shown in Fig 13, was indicated the higher conversion of CH_4_ and CO_2_ conversion at 900°C. The detection of H_2_ and CO after the blank test at 900°C indicates the decomposition methane (Eq 4). Therefore, 900°C was chosen as the appropriate temperature to conduct stability tests. Fig 13A shows the 200 h on-stream catalyst test results for the catalyst sample at 900°C. The sample showed good stability during the 200 h on-stream, and no decay of activity was found in the sample. The conversion of CH_4_ was lower than the conversion of CO_2_. The average conversion of CH_4_ and CO_2_, were 82 and 98% respectively which was reduced to. after 46 h of reaction time. Following that the conversion remained stable till it reached to 200 h. The higher the degree of the reverse water gas shift reaction process, the larger difference between CH_4_ and CO_2_ conversion, and the lower the H_2_/CO ratio. These theoretical considerations are confirmed by the actual test results shown in Fig 13A. Further the catalyst was reused in successive cycle for 200 h and the results was shown in Fig 13B. No significant reduction of CH4, CO_2_ conversion was observed.

**Fig 14:**
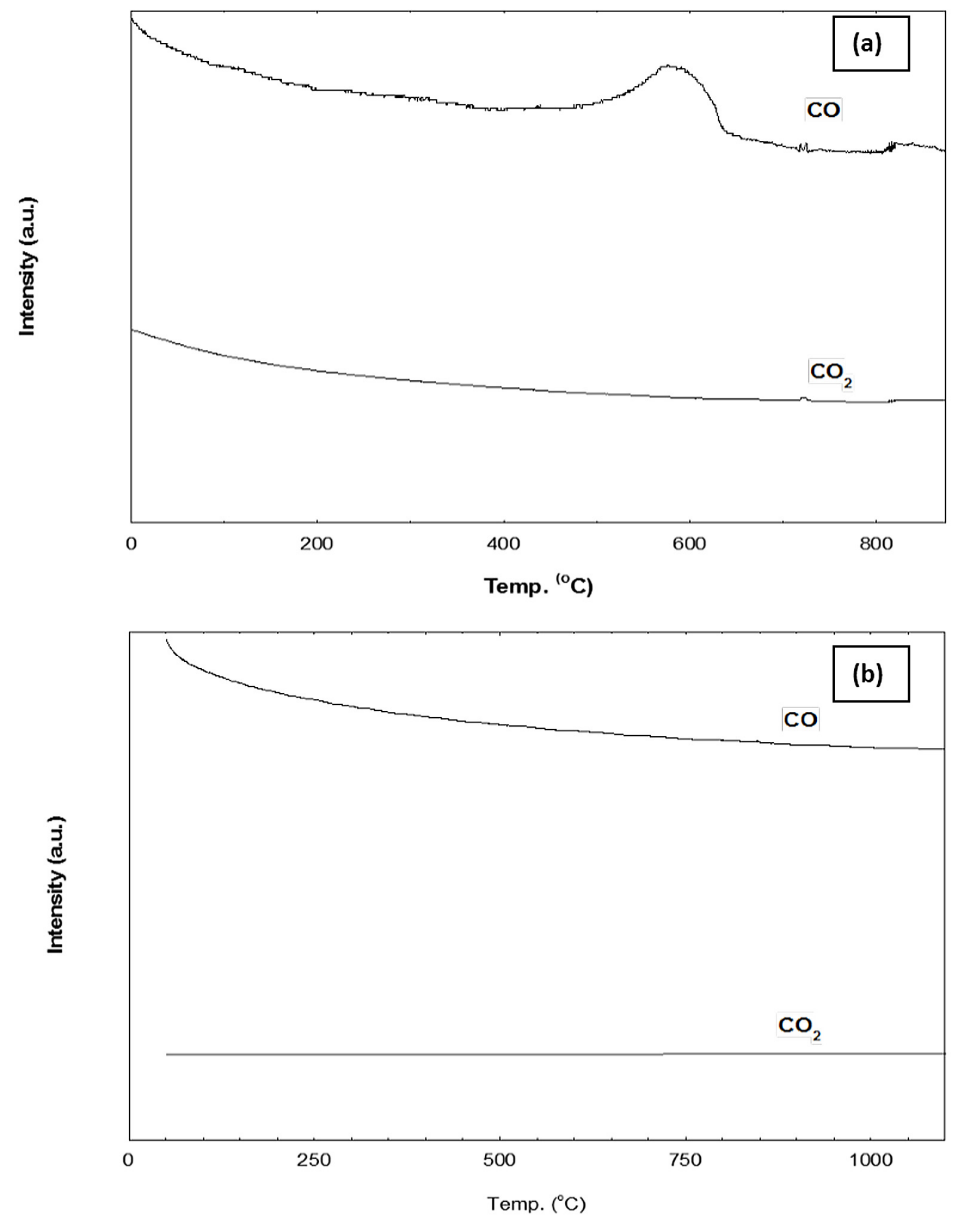
TPO curves of a) Mg_0.85_Ni_0.15_O b) Pt/Mg_0.85_Ni_0.15_O catalysts after 200h reaction with CO_2_/CH_4_ = 1:2 at 900°C.

**Fig 15:**
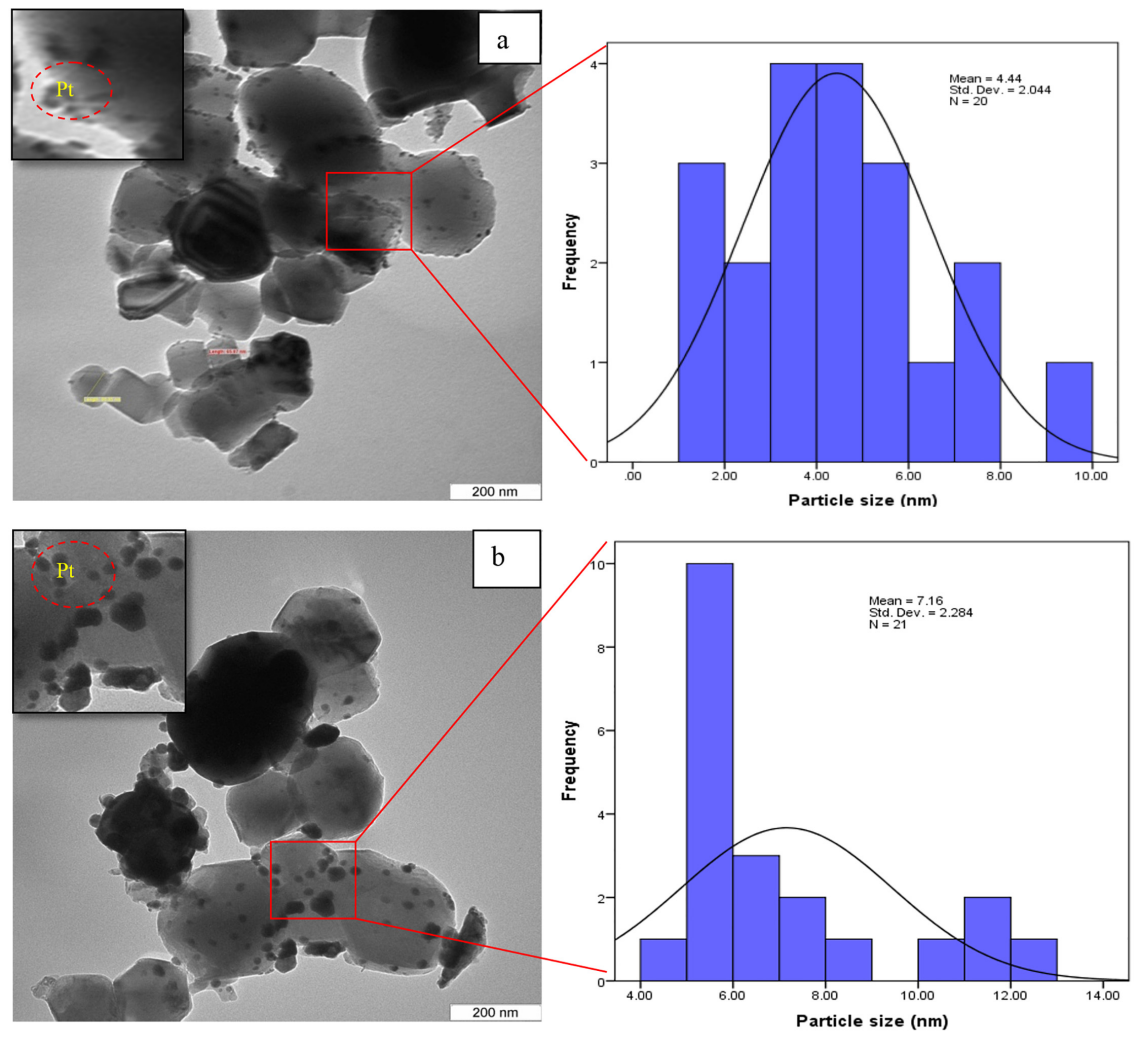
TEM analysis of (a) Pt/Mg_0.85_Ni_0.15_O catalyst; (b) Mg_0.85_Ni_0.15_O and respective Pt size distribution after reaction with CO_2_/CH_4_ = 1:2 at 900°C.

TPO-MS post-reaction test was used to evaluate coke formation on the Mg_0.85_Ni_0.15_O and Pt/Mg_0.85_Ni_0.15_O catalysts (Fig 14). The TPO profiles in Fig 14A shows the desorption at 600°C indicating the coke deposition on Mg_0.85_Ni_0.15_O catalyst surface. However, No coke deposition was observed on the Pt/Mg_0.85_Ni_0.15_O catalyst as evident from Fig 14B. TEM analysis for the spent Pt/Mg_0.85_Ni_0.15_O and Mg_0.85_Ni_0.15_O was supported the above finding represented in Fig 15. The results of TPO and TEM indicated the role of platinum metal (main catalyst) on the surface of the catalyst. However, aggregation of the Pt particle after reaction at 900°C as shown in Fig 15A and 15B, can be described by a simple Oswald ripening approach. A size-dependent morphology of the supported Pt nanoparticles may influence the ripening process in several ways. The observed growth of the projected nanoparticle areas in the TEM images depends on the nanoparticle volume, which is described in terms of the height-to-diameter ratio [30]. The nanoparticle perimeter at the Pt-oxide interface can emit or absorb diffusing species resulting in agglomeration of particle. According to Maillard et al. [30] high concentration of metal loading may results, in increase of the average diameter of isolated Pt nanoparticles

Temperature tests, as shown in Fig 12 indicated that both CH_4_ and CO_2_ conversion was increased at 900°C. However, the carbon can be deposited on the Pt metal surface, according to the following mechanism (Eqs 12–16), as proposed by Topalidis et al. [31].

$$CH_{4}+2Pt_{\left(as\right)}\;\to \;CH_{3}Pt_{\left(as\right)}\;+HPt_{\left(as\right)}$$ (12)

$$CH_{3}Pt_{\left(as\right)}+Pt_{\left(as\right)}\;\to \;CH_{2}Pt_{\left(as\right)}\;+HPt_{\left(as\right)}$$ (13)

$$CH_{2}Pt_{\left(as\right)}\;+Pt_{\left(as\right)}\;\to \;CHPt_{\left(as\right)}\;+HPt_{\left(as\right)}$$ (14)

$$CHPt_{\left(as\right)}\;+Pt_{\left(as\right)}\;\to \;CPt_{\left(as\right)}\;+HPt_{\left(as\right)}$$ (15)

$$2HPt_{\left(as\right)}\;\to \;H_{2\;\left(g\right)}\;+2Pt_{\left(as\right)}$$ (16)

The effect of NiO promoter in the catalyst on dry reforming of methane has been proposed by Nakamura et al. [32]. According to the mechanism (Eqs 17–21), CO_2_ is activated on the support in proximity to the metal particle to form a carbonate species. Following that, the carbonate is reduced by CH_x_ species to form carbon monoxide (CO).

$$CO_{2\left(g\right)}\to \;CO_{2\;\left(support\right)}$$ (17)

$$CO_{2\;\left(support\right)}\;+\;O^{2-}{}_{\left(support\right)}\;\to \;CO_{3}{{}^{2-}}_{\left(support\right)}$$ (18)

$$CO_{2\left(g\right)}\to \;CO_{2\;\left(support\right)}$$ (19)

$$CO_{3}{{}^{2-}}_{\left(support\right)}\;+\;2H_{\left(support\right)}\;\to \;HCO_{3}{{}^{2-}}_{\left(support\right)}\;+OH^{-}{\;}_{\left(support\right)}$$ (20)

$$CO_{\left(support\right)}\to \;CO_{\left(g\right)}$$ (21)

Carbon formation on the main catalyst (Pt metal) of the dry reforming of methane reaction was eliminated by the role of Ni metal as shown in S1 Fig The CO_2_ adsorption was enhanced by the presence of NiO as it increases the basicity of the catalyst. Following the adsorption, formation of carbonate species, mostly occurred on the NiO, which later dissociated to CO_2_ into CO and O. The oxygen atom then took by Ni metal on the surface reacted with the carbon deposited on the metal and produced CO [33]. When the lower concentrations of NiO, the CO_2_ conversion was increased by forming strongly ionic oxides NiCO_3_, which resulted in the attraction of CO_2_ to the catalyst surface and, therefore, increased CH_4_ conversion. When NiO was present in higher concentrations, the conversion of both CH_4_ and CO_2_ decreased. This probably happened as a result of increase in Pt electron density [34]. The NiCO_3_ species participated directly in DRM by decomposing to produce CO and providing oxygen species to react with the carbon deposited at the interface of Pt-NiCO_3_. Likewise, NiO supported catalysts can facilitate the dissociation of adsorbed CO_2_.

**Fig 16:**
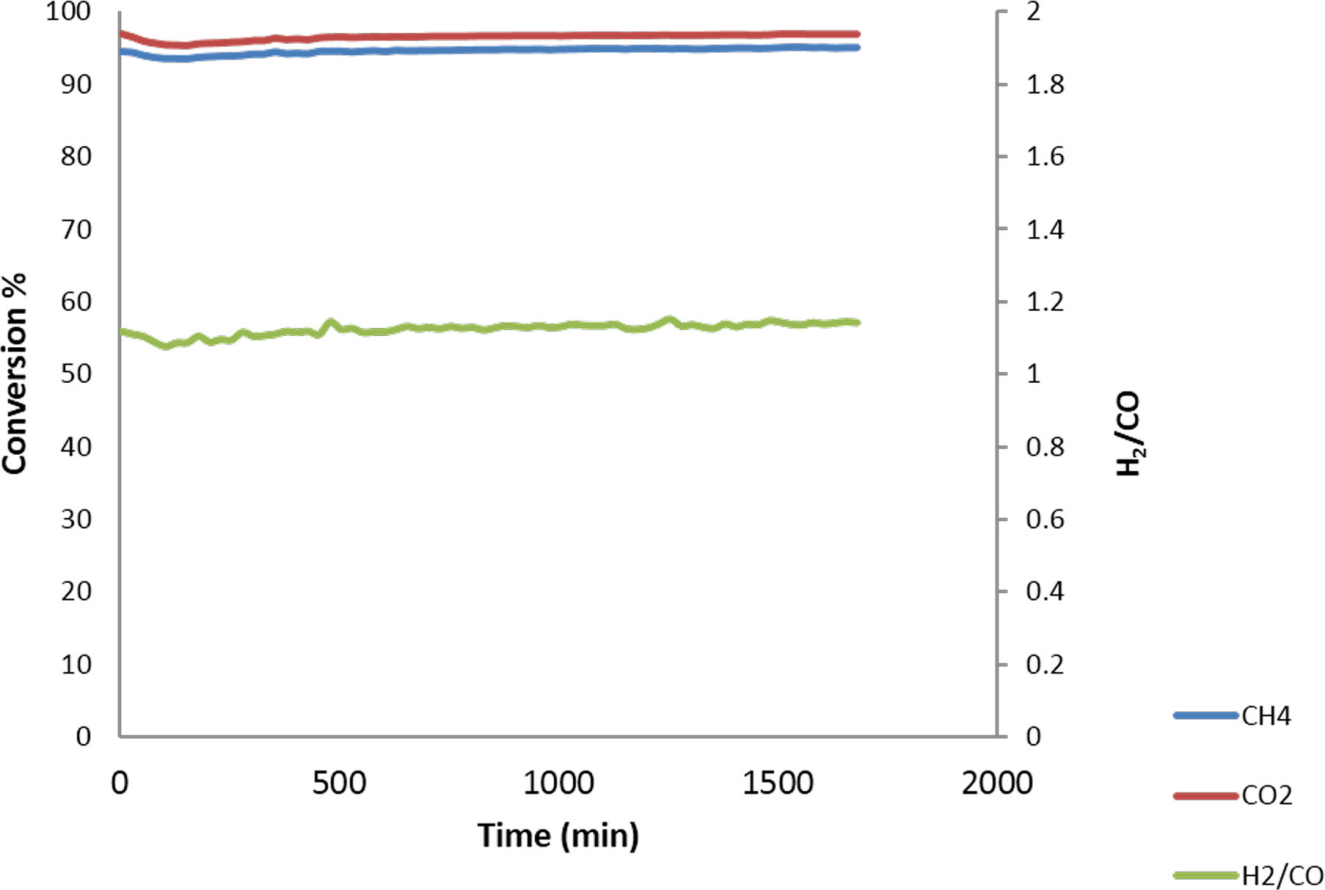
DRM reaction of the Pt/Mg_0.85_Ni_0.15_O catalyst under 900°C with 1.25% O_2_.

Dry reforming of biogas reaction can be enhanced by carried out with the presence of low concentrations of oxygen flow (1.25%). Fig 16 shows the increament of CH_4_ conversion from 82 to 95% due to adding supply of oxygen to the DRM reactant mixture [35]. However, the CO_2_ conversion and the H_2_/CO ratio were not affected might be due to the reaction of oxygen with CH_4_ to produce CO and H_2_O (Eq 22), and finally the steam reacts with the deposited carbon to give syngas according to the Eq 23. Furthermore, O_2_ can be reduced coke deposition on the catalyst (Eq 24). Dry reforming of methane has been investigated with noble (Rh, Ru, Pd and Pt) and non-noble metal (Ni, Co and Fe) based catalysts [36]. A conclusion has been by the researchers [36–38] that their superior coking resistance, higher stability and activity are especially for higher temperature applications (>750°C). Hou et al. [37] incorporated different active metals (Rh, Ru, Pt, Pd, Ir, Ni and Co) over alumina and the influence of noble metals (Rh, Ru, Pt, Pd, Ir) on the coking resistance ability of the catalyst has been examined. The catalytic activity and stability trend of the catalysts reported by Hou et al. [37] was Rh/α-Al_2_O_3_>Ru/α-Al_2_O_3_>Ir/α-Al_2_O_3_>Pd/α-Al_2_O_3_>Pt/α-Al_2_O_3_. Similarly, the catalytic activity and stability for Pd, Pt and Au catalysts as investigated by Tsyganok et al. [39] were Pd/MgAlO*x*>Pt/MgAlO*x*>Au/MgAlO*x*. It was reported that high dispersion of Pt or Au and and smaller particle size assisted in the reduction of carbon deposits, agglomeration and sintering [39]. A comparison of noble (Rh, Ru, Pd, Ir and Pt) and non-noble (Ni and Co) metal catalysts was made by Tsyganok et al. [39] and reported that the Ni and Co catalysts showed higher catalytic activities compared to the noble metal supported catalysts. However, the higher coke deposition for Ni (24.0) and Co catalysts indicate their poor coke resistance ability compared to noble metal catalysts. Thus, the deactivation arising from the coke deposition is the major obstacle in the application of Ni-based catalyst. Other workers [40] have discussed a similar phenomenon and reported that bimetallic catalyst (0.4Pt−Ni/γ-Al_2_O_3_) showed lower carbon deposition (6 wt%) compared to the monometallic (Ni/γ-Al_2_O_3_) catalyst (45 wt%). This indicates that addition of noble metals to Ni catalyst leads to the reduction of carbon deposition and produced smaller particle size compared to monometallic Ni catalysts. As a result of this process the carbon deposition is reduced and consequently the lifetime of the catalyst could be improved using Pt/Mg_1-x_Ni_x_O catalyst.

$$CH_{4}\;+\;1.5\;O_{2}\;\to CO\;+2H_{2}O_{\left(v\right)}$$ (22)

$$C_{\left(s\right)}\;+\;H_{2}O\;\to CO\;+H_{2}$$ (23)

$$C_{\left(s\right)}\;+\;O_{2}\;\to CO_{2}$$ (24)

## Conclusion

The results presented in this paper demonstrate that the addition of nickel to the support has several promotional effects. The promoters can increase the thermal stability of the Pt/Mg_1-x_Ni_x_O catalyst by stabilizing the cubic phase of magnesia.The narrow scan of Mg2p, Ni2p and Pt4f revealed that the oxide species of these metals are a mixture of MgO and Mg(OH)_2_, NiO and Ni(OH)_2_ and PtO respectively. This stabilization results in an increase in the surface area and thus, increases in the density of CO_2_ adsorption sites near the metal particle. Hence, the ability of the support to enhance the dissociation of CO_2_ near the Pt particle and the transfer of oxygen to the coked metal greatly accelerates the cleaning mechanism.These facts emphasize that the incorporation of Pt and Ni species in the catalyst prevent coke formation onto the catalyst and increase the syngas production from greenhouse gasses over Pt/Mg_1-x_Ni_x_O catalysts.

## Supporting Information

S1 Fig Effect of promoter to remove carbon from Pt. (TIF)
