# Supplementary figures and images for: High Coke-Resistance Pt/Mg1-xNixO Catalyst for Dry Reforming of Methane
Source: PLoS One. 2016 Jan 8;11(1):e0145862. doi: 10.1371/journal.pone.0145862 (PMC4706417; doi:10.1371/journal.pone.0145862)

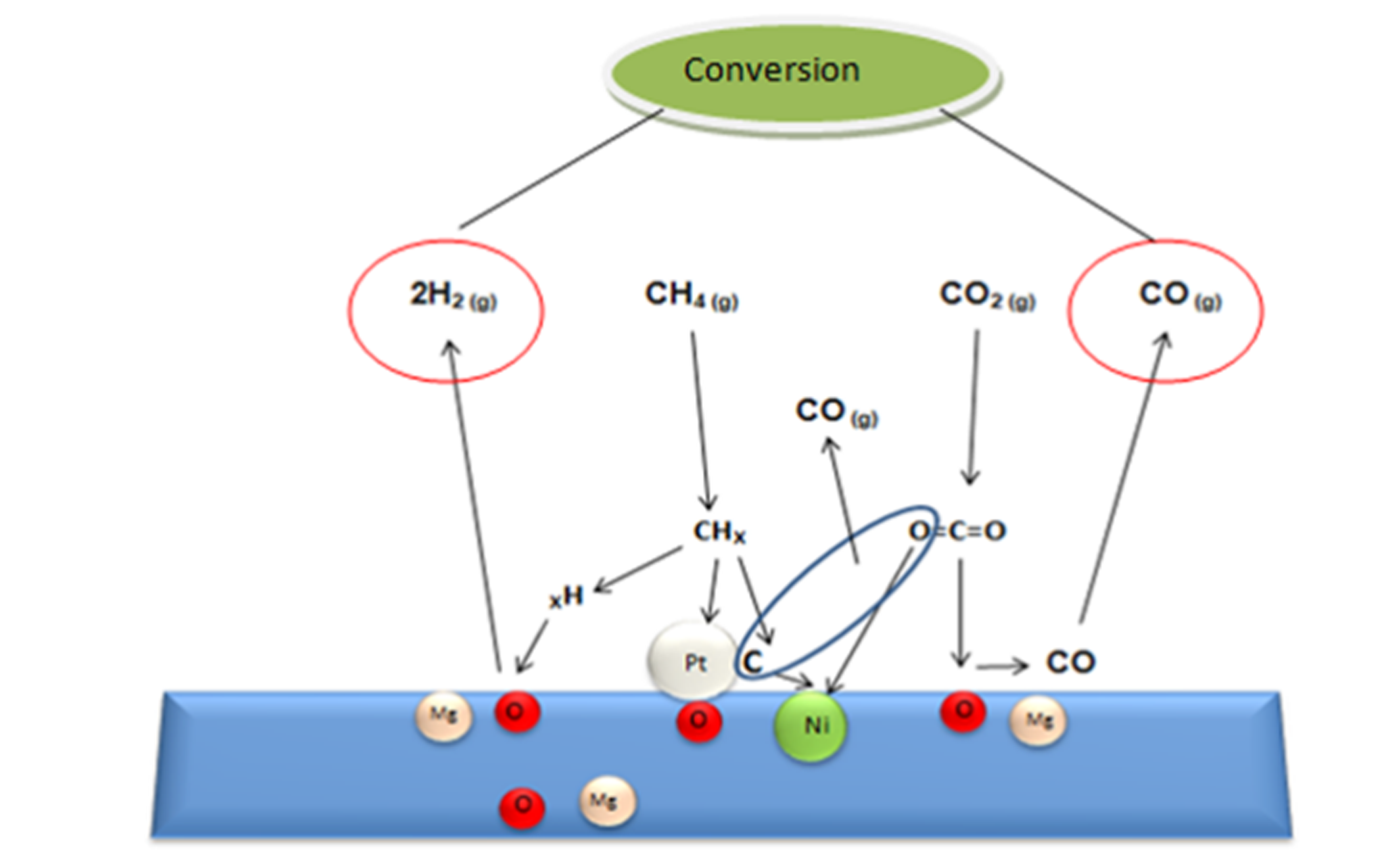

Supplement: S1 Fig — (TIF) [file pone.0145862.s001.tif]
